# Comparison of Protein B Polymerase Chain Reaction (PCR) With IS6110 PCR for Diagnosis of Tuberculous Meningitis Patients

**DOI:** 10.7759/cureus.33783

**Published:** 2023-01-15

**Authors:** Vineet Sehgal, Megha Sharma, Priyal LNU, Kusum Sharma, Aman Sharma, Navneet Sharma, Manish Modi

**Affiliations:** 1 Neurology, Amandeep Medicity, Amritsar, IND; 2 Microbiology, All India Institute of Medical Science, Bilaspur, IND; 3 Neurology, Sehgal's Neuro and Child Care Center, Amritsar, IND; 4 Neurology, Postgraduate Institute of Medical Education and Research, Chandigarh, IND; 5 Emergency Medicine, Postgraduate Institute of Medical Education and Research, Chandigarh, IND

**Keywords:** tuberculous meningitis, is6110 pcr, molecular diagnosis of infectious diseases, nucleic acid amplification, extra pulmonary tuberculosis

## Abstract

Purpose

Tuberculous meningitis (TBM) is a diagnostic challenge. With the conventional staining and culture techniques being too insensitive and time-consuming, and the commercial detection systems being costly, polymerase chain reaction (PCR) seems lucrative for routine laboratories. The purpose of this study was to evaluate the diagnostic potential of protein b antigen polymerase chain reaction (Pab PCR) for TBM, in comparison to IS6110. Another purpose was to compute a cut-off at which adenosine deaminase (ADA) could diagnose TBM.

Material and methods

This is a prospective case-control study to measure the diagnostic accuracy of PCR, BACTEC culture, Lowenstein-Jensen (LJ) culture, ADA, and acid-fast bacilli (AFB) smear tests in TBM. CSF from 50 TBM patients (10 confirmed, 40 clinically suspected) and 40 controls was subjected to Pab PCR and IS6110 PCR, and performance was compared against culture and composite reference standards.

Results

The overall sensitivity, specificity, positive predictive value (PPV), and negative predictive value (NPV) of Pab PCR in diagnosing TBM were 82%, 100%, 100%, and 81.63%, respectively, and that of IS6110 PCR were 74%, 100%, 100%, and 75.47%, respectively. Both PCRs outperformed culture (p<0.001). Though performance of both PCRs was comparable (p=0.335) with excellent agreement (k=0.86), Pab PCR detected four additional cases, one culture-positive and three culture-negative clinically suspected. ADA of 6.5 IU/L was able to differentiate between TBM and non-TBM with 86% sensitivity and 63% specificity.

Conclusions

Molecular tools such as PCR have the potential to increase the clinician's ability to diagnose tuberculous meningitis. Pab PCR is a rapid and reliable method for diagnosing TBM in routine microbiology laboratories.

## Introduction

Tuberculous meningitis (TBM) is a form of extrapulmonary TB that is frequently fatal and has devastating long-term complications [1,2]. The death rate can be as high as 44-69% in impoverished nations [3]. Prompt and accurate confirmation of TBM is necessary to improve patient outcomes. The clinical features, radiological and biochemical investigations of TBM overlap with several other infectious and non-infectious conditions of the central nervous system. Demonstrating acid-fast bacilli (AFB) in the cerebrospinal fluid (CSF) by Ziehl-Neelsen staining or isolating *Mycobacterium tuberculosis *on culture remains the "gold standard" for diagnosing TBM. Although microscopy is quick and inexpensive, it can detect AFB only when its concentration is more than 10,000 organisms per milliliter and has a fairly poor sensitivity (10-20%) [4]. Culture, another established method, is not very sensitive (less than 50%), and results are unavailable for weeks due to long generation time of 22-24 hours [5]. In the context of these shortcomings, nucleic acid amplification tests (NAAT) have emerged to enable clinicians to make accurate diagnoses promptly (approximately 3-6 hours from the receipt of the specimen) [6].

NAATs have revolutionized TBM diagnosis by amplifying a specific nucleic acid region (pathogen-specific DNA sequence) that uniquely defines *M. tuberculosis* [7]. The polymerase chain reaction (PCR) is the most well-known NAAT that consists of extracting the DNA of the pathogen from the sample, amplifying it, and detecting it. Several *M. tuberculosis*-specific gene targets have been explored in the past for the diagnosis of TBM, including MPB-64, 65 kDa antigen, IS6110 insertion sequence, IS986, protein b antigen (Pab), and 16S rRNA [8-11]. The Pab gene codes for a 38 kDa secretory protein of *M. tuberculosis*. The current study aimed to evaluate the diagnostic potential of Pab gene for the diagnosis of TBM. Its performance was compared against IS6110 PCR and conventional methods like Ziehl-Neelsen (ZN) smear and culture. The clinical and biochemical profile of the patients was also noted and performance of adenosine deaminase (ADA) in cerebrospinal fluid (CSF) was also evaluated.

## Materials and methods

Study design

This prospective case-control study was carried out at a tertiary care hospital in North India. Participants included attendees of outpatient and inpatient settings at neurology department and emergency department of the institute, and samples were processed in the department of microbiology. The institutional ethics committee approved the study.

Study population

Adult patients presenting at our tertiary care center with fever associated with headache and vomiting with/without altered sensorium were included. After taking informed consent from the patient, detailed clinical history was taken. The clinical evaluation was done looking for signs of meningeal irritation, raised intracranial pressure, cranial nerve deficits, focal neurological deficits, and other systems for evidence of tuberculosis elsewhere in the body. Patients with TBM were clinically divided into three stages based on severity using the British Infection Society guidelines [12]. Routine blood tests and radiological investigations were carried out and staging of TBM was done as per British Infection Society guidelines [12]. All the information was recorded on a proforma. The pregnant patients were excluded. The management of patients was not governed by the results of microbiological investigations and patients were followed up for a period of 12-18 months.

Study groups

The Marais criteria, incorporating clinical, biochemical, radiological, and microbiological aspects for a uniform case definition of TBM, was used to divide the study population into the following three groups [13]. Group 1 - confirmed TBM (n=10), patients with microbiological evidence of *M. tuberculosis *in smear or culture. Group 2 - clinically-suspected TBM (n=40), consisting of patients that were culture/smear-negative but fulfilled other criteria, i.e., a score of >10-12 on Marais diagnostic criteria depending on availability of radiological evidence [13]. Group 3 - control patients (n=40), consisting of patients with confirmed meningitis by agents other than TB, such as bacterial, viral and fungal meningitis (n=20), and patients of non-infectious neurological conditions such as motor neuron disease, Guillain-Barre syndrome, dementia, neuropathy, transverse myelitis, etc. (n=20).

Sample collection and distribution

Lumbar puncture was used to collect CSF aseptically. Two to three milliliters of CSF was sent for microbiological and biochemical examination (proteins, sugar, total and differential cell count, and ADA). The microbiology lab handled each sample using mycobacteriological safeguards. A 1.5-2 mL pellet was obtained by centrifuging the sample at 3000 g for 15 minutes. It was homogenized by voxtering and distributed as follows: 0.1 mL for ZN staining, 0.1 mL for Gram staining, 0.2 mL for Lowenstein-Jensen (LJ) medium culture, 0.5 mL for BACTEC MGIT 960 (BD, Franklin Lakes, NJ) culture, and 0.4 mL for PCR. The remainder was cryopreserved. Case-by-case CSF tests included venereal disease research laboratory (VDRL) test, Indian ink staining, and cryptococcal latex agglutination. Each sample was coded to blind the researcher to its initial category.

Sample processing

Standard ZN staining revealed pink or red slender, beaded acid-fast bacilli (AFB). A 0.1 mL of deposit was inoculated on two LJ slants and cultured for eight weeks at 37°C, before reporting negative. Any growth was smeared and ZN-stained to confirm AFB. A 0.5 mL of concentrated CSF was inoculated and monitored for BACTEC. Any beeping bottle was tested with ZN stain. DNA was extracted using commercially available Qiagen DNA extraction kit (QIAGEN, Hilden, Germany) and stored at -80°C till use.

PCR

The extracted DNA was subjected to two sets of PCR, one for Pab and another for IS6110, using previously defined protocols and primer sequences [8,14]. With each run, positive (H37Rv strain) and negative control (double distilled water) were used to ensure validity. After DNA amplification, the samples were run on 1.5% agarose gel electrophoresis stained with ethidium bromide along with a 100 bp ladder and then examined under UV. A positive result was noted if bands were visualized at 419 bp for Pab gene and 123 bp for IS6110 gene.

The following primers were used to amplify a 419 bp sequence present in the 38 kDa protein antigen b (Pab) gene as per the protocol described previously: forward primer Pabf - 5′-ACCACCGAGCGGTTCGCCTGA-3′ and reversed primer Pabr - 5′-GATCTGCGGGTCGTCCCAGGT-3′ [14]. DNA amplification of the 123 bp IS6110 insertion element was carried out by following two oligonucleotide primers sequence: IS6110f 5′-CCT GCG AGC GTA GGC GTC GG-3′ and IS6110 r 5′- CTC GTC CAG CGC CGC TTC GG-3′.

Reference standards

The performance of Pab PCR and IS6110 PCR was evaluated using following two reference standards: culture for confirmed TBM and composite reference standard (CRS) for clinically-suspected TBM. The CRS incorporated clinical, microbiological, and radiological features as described previously [15].

Statistical analysis

Each test's sensitivity, specificity, and positive and negative predictive values were analyzed using conventional formulas and provided with 95% CI. Categorical data were compared using chi-square with Yates adjustment, while numerical data were compared using Student's t-test and Mann-Whitney test. A receiver operating curve (ROC) was used to determine the best ADA cut-off for separating patients from controls. Its effectiveness was measured by area under the curve (AUC). Cohen's kappa was utilized to determine agreement between PCR and reference standard. GraphPad Prism was used for calculation (GraphPad Inc., La Jolla, CA). P<0.05 was considered statistically significant.

## Results

Profile of study population

Demographical and Clinical Profile

The mean age of TBM cases and control group was 35.14±18.53 and 40.20±17.52 years, respectively. Men constituted 54% patients in TBM group and 55% in control group. There was no statistically significant difference between the two groups with reference to age and sex. The symptoms and radiological profiles of study group are presented in Table 1. Eleven (22%) patients were in stage I of TBM, 23 (46%) in stage II, and 16 (32%) in stage III at the time of first visit. Control group was comprised of patients with pyogenic meningitis (n=14), viral encephalitis (n=3), fungal meningitis (n=3), and non-infectious neurological disorders (n=20).

**Table 1:** Clinical characteristics of patients in TBM group at presentation. TBM: tuberculous meningitis

Symptoms/features	N (%)
Fever	49 (98%)
Headache	41 (82%)
Triad (fever+headache+vomiting)	30 (60%)
Seizures	13 (26%)
Coma	12 (24%)
Cranial nerve involvement	26 (52%)
Bilateral disc edema	6 (12%)
Hemiparesis	5 (10%)
Radiological features	
Hydrocephalus	17 (34%)
Basal exudates	11 (22%)
Infarcts	5 (10%)
Tuberculomas at presentation	4 (8%)
Evidence of extraneural tuberculosis	
Pulmonary	7 (14%)
Tuberculous lymphadenitis	2 (4%)
Abdominal tuberculosis	1 (2%)

Biochemical Profile

**Figure 1:**
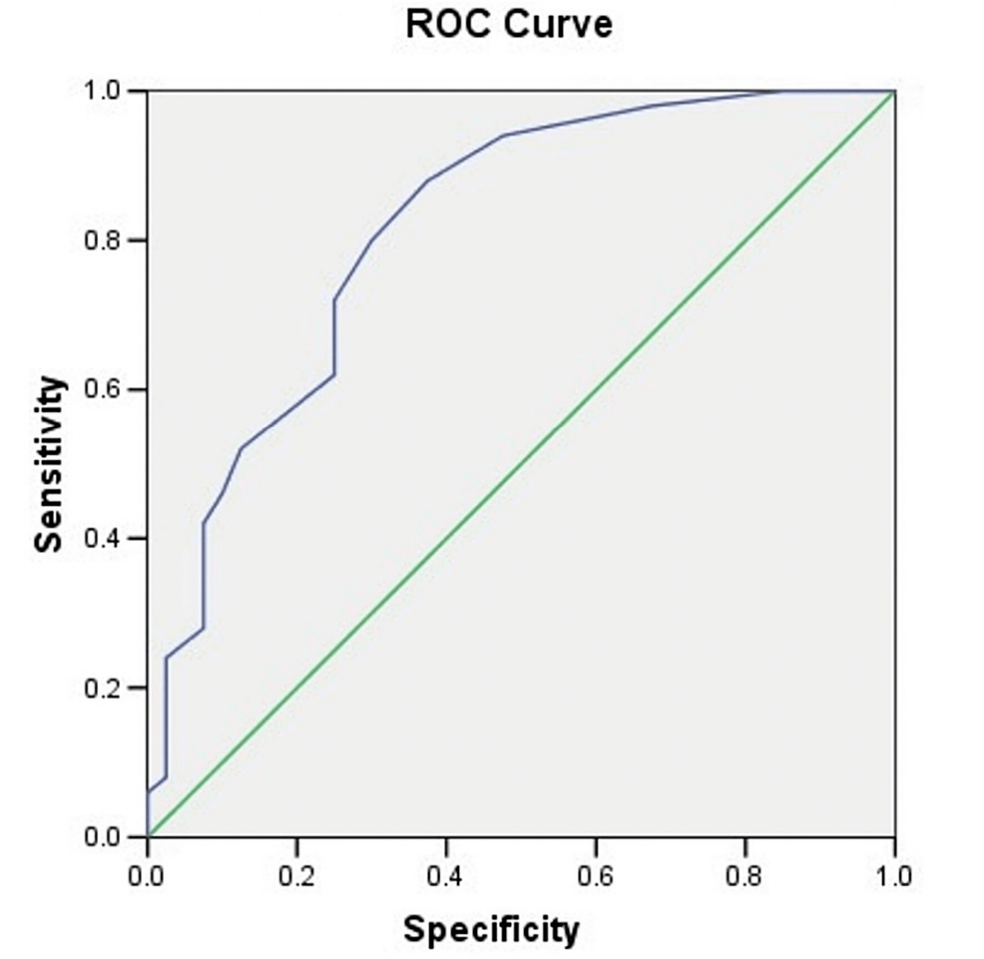
Receiver operating characteristic curve of the adenosine deaminase (IU/L) levels calculated in the CSF. ROC: receiver operating curve

Mean CSF ADA in TBM patients was 12.82±5.83 IU/L, substantially higher than the control group (6.97±4.28 IU/L) (p<0.001) and infectious meningitis subgroup (9.15±4.92 IU/L) (p=0.014). No significant difference was seen between TBM and pyogenic meningitis (10.5±5.08 IU/L) (p=0.225) and between confirmed (16.0±7.28 IU/L) and suspected TBM (12.02±5.22 IU/L) (p=0.111). ADA levels increased between TBM stages I and III (p=0.010) and II and III (p=0.032). The ROC curve demonstrated that the optimum cut-off value for CSF ADA activity to differentiate TBM from non-TBM was 6.5 IU/L, with diagnostic accuracy of 76%, AUC of 0.817, and sensitivity and specificity of 88% and 63%, respectively (Figure 1).

Microbiological profile of the study population

Results of microbiological investigations in diagnosing TBM-ZN smear, LJ culture, BACTEC culture, IS6110 PCR and Pab PCR were positive in 4 (8%), 7 (14%), 10 (20%), 37 (74%), and 41 (82%) patients, respectively, of 50 TBM cases. Control group tests were negative. Overall, the sensitivity, specificity, PPV, and NPV of Pab PCR were 82%, 100%, 100%, and 81.63%, and that of IS6110 PCR was 74%, 100%, 100%, and 75.47%, respectively (Table 2). PCR testing exhibited higher positivity than conventional tests and BACTEC culture (p<0.001). Both PCRs performed similarly (p=0.335).

**Table 2:** Diagnostic accuracy of different techniques used for the diagnosis of TBM. PPV: positive predictive value; NPV: negative predictive value; PCR: polymerase chain reaction; LJ: Lowenstein-Jensen; ZN: Ziehl-Neelsen

Tests	Test results	TBM groups (n=50)	Control group (n=40)	Sensitivity (%)	Specificity (%)	PPV (%)	NPV (%)
Pab PCR	Positive	41	0	82 (68.5-91.4%)	100% (91.1-100%)	100%	81.63% (71.1-88.9%)
	Negative	9	40				
IS6110 PCR	Positive	37	0	74% (59.6-85.3%)	100% (91.1-100%)	100%	75.47% (65.8-83%)
	Negative	13	40				
BACTEC culture	Positive	10	0	20% (10-33.7%)	100% (91.1-100%)	100%	50% (46.5-53.4%)
	Negative	40	40				
LJ culture	Positive	7	0	14% (5.8-26.7%)	100% (91.1-100%)	100%	48.19% (45.4-50.9%)
	Negative	43	40				
ZN smear	Positive	4	0	8% (2.2-19.2%)	100% (91.1-100%)	100%	46.5% (44.4-48.5%)
	Negative	46	40				

Performance of Pab PCR and IS6110 PCR in diagnosing subgroups and stages of TBM are as follows: confirmed TBM - out of 10 confirmed cases of TBM, Pab PCR detected *M. tuberculosis *in all 10 cases while IS6110 PCR detected nine cases, thus yielding a sensitivity of 100% and 90%, respectively, for Pab and IS6110 against culture. Suspected cases - out of 40 clinically-suspected TBM cases, Pab PCR detected *M. tuberculosis *in 31 cases while IS6110 PCR detected 28 cases, thus yielding a sensitivity of 77.5% and 70%, respectively, for Pab and IS6110 against CRS (Table 3).

**Table 3:** Positivity rate of different techniques used for the diagnosis of TBM. The positivity rate is depicted as N (%). TBM: tuberculous meningitis; PCR: polymerase chain reaction; LJ: Lowenstein-Jensen; Pab: protein b antigen

Group	Subgroup	Number of patients	Smear positive	LJ culture positive	BACTEC culture positive	IS6110 PCR positive	Pab PCR positive
Group 1	Confirmed TBM (culture positive)	10	4 (40%)	7 (70%)	10 (100%)	9 (90%)	10 (100%)
Group 2	Clinically suspected TBM (culture negative)	40	0	0	0	28 (75%)	31 (77.5%)
Total		50	4 (8%)	7 (14%)	10 (20%)	37 (74%)	41 (82%)
Group 3	Non-TBM infectious meningitis group	40	-	-	-	-	-

The sensitivity of both PCRs increased with the stage of TBM, but the difference was not statistically significant (p=0.614). Comparison of Pab PCR and IS6110 PCR is as follows: 36 cases were positive and 49 were negative by both PCR probes. The two tests gave concordant results in 85 out of 90 samples with an excellent agreement between the two methods, kappa=0.865.

Cost and time comparison of different tests is as follows: the average turnaround time, from receiving CSF sample to final report, was 21 days for LJ culture, 10 days for BACTEC culture, 2 hours for ZN smear, and 4 hours for PCR. The average cost, excluding the cost of personnel and machine, was $0.5 for ZN smear, $1 for LJ culture, $2 for PCR, and $5 for BACTEC.

## Discussion

The present study was conducted to analyze and compare the diagnostic accuracy of ZN smear for AFB, LJ culture, BACTEC MGIT 960, Pab PCR, and IS6110 PCR in diagnosing TBM. The mean age of TBM patients was 35.14±18.53 years and 54% were males. These findings were comparable to previous studies reported among TBM patients [3,16-17]. The most common symptoms at the time of presentation, in the present study, were fever (n=49, 98%) and headache (n=41, 82%); and the triad of fever, headache, and vomiting was present in 30 (60%) patients. This incidence of fever, headache, and vomiting was higher in the present study as compared to previous studies [3,18]. This may be due to the mandatory inclusion criteria of fever and headache in the present study group and the tertiary care nature of our institute. The incidence of seizures, hemiparesis, cranial nerve abnormalities; and radiological features was comparable with previous studies [3;18]. Eventually, 31 (62%) patients in TBM group responded well to anti-tubercular therapy, while 11 (22%) died and remaining eight (16%) were lost to follow-up.

In the present study, the mean CSF ADA value in TBM patients was 12.82±5.83 IU/L, which was significantly higher than the control group, 6.97±4.28 IU/L (p<0.001), but comparable with pyogenic meningitis subgroup. In the present study, a cut-off of 6.5 IU/L was able to differentiate between TBM and non-TBM groups with a sensitivity and specificity of 88% and 63%, respectively. Our findings are concordant with those of Raviraj et al. who reported that a cut-off of 6.65 IU/L was able to differentiate between TBM and non-TBM groups with a sensitivity and specificity of 85.3% and 84.3%, respectively [19]. Prasad et al. could differentiate between TBM and acute bacterial meningitis by using a higher cut-off of 10 IU/L that gave a sensitivity and specificity of 68.3% and 92.7%, respectively [20]. The difference among these studies could be due to the different methodologies used and presence of co-infections. The meta-analysis involving >3400 CSF samples also could not establish a single cut-off value to differentiate between different types of meningitis, thereby proving dubious nature of CSF ADA in diagnosing TBM [20,21].

Pab PCR had 82% sensitivity in detecting TBM in the current study. The previously reported sensitivity of Pab PCR ranges from 42.8% to 82.8% [8,22]. Pab PCR correctly diagnosed all 10 culture-positive TBM cases in the current study. This was similar to Sjöbring et al. where all four culture-positive cases were positive by Pab PCR and better than Sharma et al. where one culture-positive case was missed [8,22]. Pab PCR detected 77.5% of clinically-suspected TBM cases, in the current study, which lies within the reported range of 75-81.7% [8,23]. These subtle differences among studies, despite using same primers for amplification, could be due to the difference in CSF processing and volume and DNA extraction protocols.

Pab PCR had a higher sensitivity than IS6110 PCR (82% vs 74%), however, the difference was not statistically significant. Both PCRs agreed well. Negi et al. observed that Pab PCR was equivalent to IS6110, 85 B mRNA, and 65kDa antigen (p>0.05) among 172 pulmonary and extrapulmonary specimens, including five CSF [14]. Both Pab and IS6110 PCRs were 100% specific in this investigation. Previous studies indicated 100% specificity with Pab PCR, while IS6110 ranged from 38% to 75% to 100% [8,24-26].

Pab PCR detected four cases that IS6110 PCR missed. One was culture-positive and three culture-negative. IS6110, a multi-copy gene with up to 20 copies, is the most favored target for *M. tuberculosis*, however, 15-40% of North Indian *M. tuberculosis *isolates may have only one copy of IS6110 [27]. Possibly, these four cases lacked IS6110 gene. Multiplexing PCR incorporates different genes' benefits. Most studies have shown improved sensitivity and specificity by employing multiple gene targets for TBM PCR, notably Pab, MPB64, and IS6110 and IS6110 and TRC4, however, Bhigjee et al. did not report many benefits using IS6110, MPB64, and PT8/9 [28,29]. These discrepancies may be due to differences in *M. tuberculosis *strains, PCR techniques, and CSF sample processing [30].

Limitations of the study

First, just two PCR targets were examined in a limited sample, because trial time was limited, and participants were followed for 12-18 months. Second, patient outcomes were only based on clinical stage of TBM, not drug susceptibility, *M. tuberculosis *lineage, or HIV status [31]. Third, only conventional PCR was used, other NAATS like nested and real-time PCR, commercial assays like GeneXpert, GeneXpert Ultra, and Truenat, or metagenomics next-generation gene sequencing (mNGS), are also available [32-36]. Fourthly, drug resistance was not detected, though MDR and XDR TBM has been reported and simultaneous reporting is suggested [37-40]. Lastly, Pab PCR cannot differentiate between live and dead bacilli, for which mRNA is needed [41].

## Conclusions

The present study showed that Pab PCR is a good and rapid method for the diagnosis of TBM. It showed the highest sensitivity (82%) as compared to other tests, including PCR IS6110 and detected additional four cases that were missed by IS6110. Thus, Pab PCR can be used as a simple, cheap, fast, and reliable test to improve the clinician's ability to detect TBM.

## References

[1] Aggarwal A, Mehta S, Gupta D, Sheikh S, Pallagatti S, Singh R, Singla I (2012). Dental students' motivations and perceptions of dental professional career in India. J Dent Educ.

[2] Wilkinson RJ, Rohlwink U, Misra UK (2017). Tuberculous meningitis. Nat Rev Neurol.

[3] Brancusi F, Farrar J, Heemskerk D (2012). Tuberculous meningitis in adults: a review of a decade of developments focusing on prognostic factors for outcome. Future Microbiol.

[4] Stadelman AM, Ssebambulidde K, Buller A (2022). Cerebrospinal fluid AFB smear in adults with tuberculous meningitis: a systematic review and diagnostic test accuracy meta-analysis. Tuberculosis (Edinb).

[5] Cresswell FV, Davis AG, Sharma K (2021). Recent developments in tuberculous meningitis pathogenesis and diagnostics. Wellcome Open Res.

[6] Bahr NC, Meintjes G, Boulware DR (2019). Inadequate diagnostics: the case to move beyond the bacilli for detection of meningitis due to Mycobacterium tuberculosis. J Med Microbiol.

[7] Ssebambulidde K, Gakuru J, Ellis J, Cresswell FV, Bahr NC (2022). Improving technology to diagnose tuberculous meningitis: are we there yet?. Front Neurol.

[8] Sharma K, Sharma A, Singh M (2010). Evaluation of polymerase chain reaction using protein b primers for rapid diagnosis of tuberculous meningitis. Neurol India.

[9] Thierry D, Brisson-Noël A, Vincent-Lévy-Frébault V, Nguyen S, Guesdon JL, Gicquel B (1990). Characterization of a Mycobacterium tuberculosis insertion sequence, IS6110, and its application in diagnosis. J Clin Microbiol.

[10] Bhattacharya B, Karak K, Ghosal AG (2003). Development of a new sensitive and efficient multiplex polymerase chain reaction (PCR) for identification and differentiation of different mycobacterial species. Trop Med Int Health.

[11] Cormican MG, Barry T, Gannon F, Flynn J (1992). Use of polymerase chain reaction for early identification of Mycobacterium tuberculosis in positive cultures. J Clin Pathol.

[12] Thwaites G, Fisher M, Hemingway C, Scott G, Solomon T, Innes J (2009). British Infection Society guidelines for the diagnosis and treatment of tuberculosis of the central nervous system in adults and children. J Infect.

[13] Marais S, Thwaites G, Schoeman JF (2010). Tuberculous meningitis: a uniform case definition for use in clinical research. Lancet Infect Dis [Internet.

[14] Negi S, Anand R, Pasha S, Gupta S, Basir SF, Khare S, Lal S (2007). Diagnostic potential of Is6110, 38Kda, 65Kda, and 85B sequence-based polymerase chain reaction in the diagnosis of Mycobacterium tuberculosis in clinical samples. Indian J Med Microbiol.

[15] Vadwai V, Boehme C, Nabeta P, Shetty A, Alland D, Rodrigues C (2011). Xpert MTB/RIF: a new pillar in diagnosis of extrapulmonary tuberculosis?. J Clin Microbiol.

[16] Evans EE, Avaliani T, Gujabidze M (2022). Long term outcomes of patients with tuberculous meningitis: the impact of drug resistance. PLoS One.

[17] Chaidir L, Annisa J, Dian S (2018). Microbiological diagnosis of adult tuberculous meningitis in a ten-year cohort in Indonesia. Diagn Microbiol Infect Dis.

[18] Verdon R, Chevret S, Laissy JP, Wolff M (1996). Tuberculous meningitis in adults: review of 48 cases. Clin Infect Dis.

[19] Raviraj Raviraj, Henry RA, Rao GG (2017). Determination and validation of a lower cut off value of cerebrospinal fluid adenosine deaminase (CSF-ADA) activity in diagnosis of tuberculous meningitis. J Clin Diagn Res.

[20] Prasad PL, Chauhan S, Khurana B (2018). Evaluation of CSF ADA level in diagnosis of tubercular meningitis. Int J Contemp Med.

[21] Ekermans P, Dusé A, George J (2017). The dubious value of cerebrospinal fluid adenosine deaminase measurement for the diagnosis of tuberculous meningitis. BMC Infect Dis.

[22] Sjöbring U, Mecklenburg M, Andersen AB, Miörner H (1990). Polymerase chain reaction for detection of Mycobacterium tuberculosis. J Clin Microbiol.

[23] Shankar P, Manjunath N, Mohan K, Prasad K, Behari M, Shriniwas Shriniwas, Ahuja GK (1991). Rapid diagnosis of tuberculous meningitis by polymerase chain reaction. Lancet.

[24] Lee BW, Tan JA, Wong SC (1994). DNA amplification by the polymerase chain reaction for the rapid diagnosis of tuberculous meningitis. Comparison of protocols involving three mycobacterial DNA sequences, IS6110, 65 kDa antigen, and MPB64. J Neurol Sci.

[25] Deshpande PS, Kashyap RS, Ramteke SS, Nagdev KJ, Purohit HJ, Taori GM, Daginawala HF (2007). Evaluation of the IS6110 PCR assay for the rapid diagnosis of tuberculous meningitis. Cerebrospinal Fluid Res.

[26] Rafi W, Venkataswamy MM, Nagarathna S, Satishchandra P, Chandramuki A (2007). Role of IS6110 uniplex PCR in the diagnosis of tuberculous meningitis: experience at a tertiary neurocentre. Int J Tuberc Lung Dis.

[27] Chauhan DS, Sharma VD, Parashar D (2007). Molecular typing of Mycobacterium tuberculosis isolates from different parts of India based on IS6110 element polymorphism using RFLP analysis. Indian J Med Res.

[28] Kusum S, Aman S, Pallab R (2011). Multiplex PCR for rapid diagnosis of tuberculous meningitis. J Neurol.

[29] Narayanan S, Parandaman V, Narayanan PR, Venkatesan P, Girish C, Mahadevan S, Rajajee S (2001). Evaluation of PCR using TRC(4) and IS6110 primers in detection of tuberculous meningitis. J Clin Microbiol.

[30] Bhigjee AI, Padayachee R, Paruk H, Hallwirth-Pillay KD, Marais S, Connoly C (2007). Diagnosis of tuberculous meningitis: clinical and laboratory parameters. Int J Infect Dis.

[31] Sharma K, Modi M, Sharma M (2018). Effect of Mycobacterium tuberculosis lineage, drug resistance and HIV status on the outcome of patients with tuberculous meningitis. J Clin Diagn Res.

[32] Takahashi T, Nakayama T, Tamura M (2005). Nested polymerase chain reaction for assessing the clinical course of tuberculous meningitis. Neurology.

[33] Sharma K, Sharma M, Singh S, Modi M, Sharma A, Ray P, Varma S (2017). Real-time PCR followed by high-resolution melting curve analysis: a rapid and pragmatic approach for screening of multidrug-resistant extrapulmonary tuberculosis. Tuberculosis (Edinb).

[34] Sharma K, Sharma M, Chaudhary L (2018). Comparative evaluation of Xpert MTB/RIF assay with multiplex polymerase chain reaction for the diagnosis of tuberculous meningitis. Tuberculosis (Edinb).

[35] Sharma K, Sharma M, Shree R (2020). Xpert MTB/RIF ultra for the diagnosis of tuberculous meningitis: a diagnostic accuracy study from India. Tuberculosis (Edinb).

[36] Sharma K, Sharma M, Modi M (2021). Comparative analysis of Truenat™ MTB Plus and Xpert(®) Ultra in diagnosing tuberculous meningitis. Int J Tuberc Lung Dis.

[37] Ramachandran PS, Ramesh A, Creswell FV (2022). Integrating central nervous system metagenomics and host response for diagnosis of tuberculosis meningitis and its mimics. Nat Commun.

[38] Sharma K, Sharma M, Modi M, Goyal M, Sharma A, Ray P (2021). A decade of drug-resistant tuberculous meningitis: a wake-up call for patient-centric therapy. Indian J Med Microbiol.

[39] Sharma K, Sharma M, Modi M (2021). Heading toward resistance, headon: a case of XDR tuberculous meningitis. Neurol India.

[40] (2021). WHO consolidated guidelines on tuberculosis: module 3: diagnosis: rapid diagnostics for tuberculosis detection, 2021 update. https://www.who.int/publications/i/item/9789240029415.

[41] Sharma K, Gupta A, Sharma M, Singh S, Sharma A, Singh R, Gupta V (2022). Detection of viable Mycobacterium tuberculosis in ocular fluids using mRNA-based multiplex polymerase chain reaction. Indian J Med Microbiol.

